# Experimental and experiential recommendations for using the GreenFeed systems to measure gas flux in grazing and confined cattle

**DOI:** 10.1016/j.mex.2025.103667

**Published:** 2025-10-05

**Authors:** E.A. French, M.R. Beck, K.F. Kalscheur, D.M. Jaramillo, J.D. Derner, C.A. Moffet, B.W. Neville, K.J. Soder, R.C. O’Connor, J.A. Koziel, P. Vadas, S. Moeller, S.A. Gunter, USDA-Agricultural Research Service, Enteric Methane Intervention Team (EMIT) Working Group

**Affiliations:** a USDA Agricultural Research Service, U.S. Dairy Research Forage Research Center, Madison, WI, United States; b Texas A&M University Department of Animal Science, College Station, TX, United States; c USDA Agricultural Research Service, Center for Agricultural Resources Research, Rangeland Resources & Systems Research Unit, Fort Collins, CO, United States; d USDA Agricultural Research Service, Oklahoma and Central Plains Agricultural Research Center, El Reno, OK, United States; e USDA Agricultural Research Service, U.S. Meat Animal Research Center, Clay Center, NE, United States; f USDA Agricultural Research Service, Pasture Systems and Watershed Management Research Unit, University Park, PA, United States; g USDA Agricultural Research Service, Range and Meadow Management Research, Burns, OR, United States; h USDA Agricultural Research Service, Livestock Nutrient Management Research, Bushland, TX, United States; i USDA Agricultural Research Service, Natural Resources and Sustainable Agricultural Systems, Beltsville, MD, United States; j USDA Agricultural Research Service, Animal Production and Protection, Beltsville, MD, United States

**Keywords:** Automatic head respiration chamber, Animal efficiency, Enteric methane, Livestock production, Ruminant, Animal nutrition

## Abstract

Improving ruminant production efficiency is contingent on accurately measuring gas fluxes from individual animals in grazing and confined feeding environments The GreenFeed system (GFS) has been increasingly utilized by researchers to measure gas flux of ruminants in their production environment. However, there are wide inconsistencies in methodologies from laboratory-to-laboratory. The objective of this manuscript is to provide standardized recommendations for measuring individual animal gas fluxes of methane, carbon dioxide, oxygen, and hydrogen by the GFS, which are based on experiential and experimental evidence. The method includes:

• GFS management: Setup, maintenance, and calibration of sensors
• Animal Recommendations: Number of animals to sample, training on use of the system, and bait feeds – including type, composition, and mass. Further, we address operational considerations of using the GFS in extensive grazing environments and confined feeding operations.
• Data recommendations: pre-processing data, data cleaning, handling outliers, approaches for estimating individual animal gas fluxes, and uses of application performance interfaces in conjunction with R for statistical analyses.

Increasing standardization in GFS management across experiments and laboratory groups is greatly needed. We hope that these recommendations based on our collective experience and experimental evidence will aid in the standardization of GFS methodologies.

## Graphical abstract

**Figure:**
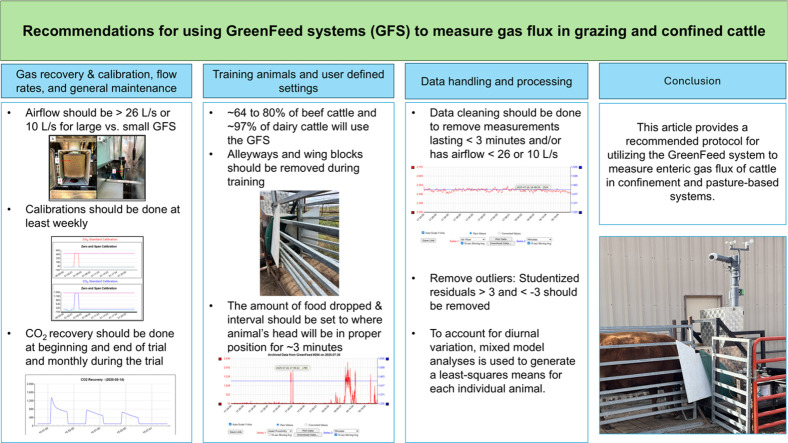

## Specifications table

**Table:** 

**Subject area**	
**More specific subject area**	Automated head chamber to measure and analyze gas fluxes in ruminant animals for production and feed efficiency
**Name of your method**	GreenFeed system best practices for measuring methane, carbon dioxide, oxygen, and hydrogen in grazing and confined cattle
**Name and reference of original method**	Gunter, S. A., and M. R. Beck. 2018. Measuring the respiratory gas exchange by grazing cattle using an automated, open-circuit gas quantification system. Transl. Anim. Sci. 2:11–18. doi:10.1093/tas/txx009. Hristov, A. N., J. Oh, F. Giallongo, T. Frederick, H. Weeks, P. R. Zimmerman, M. T. Harper, R. A. Hristova, R. S. Zimmerman, and A. F. Branco. 2015. The use of an automated system (GreenFeed) to monitor enteric methane and carbon dioxide emissions from ruminant animals. J. Vis. Exp. 2015:1–8. doi:10.3791/52,904.
**Resource availability**	C-Lock, Inc. GreenFeed large ruminant or small ruminant system to reproduce your method (e.g., equipment, data, software, hardware, reagents).

## Background

Measuring gas fluxes from ruminant livestock is an important component of metabolism research with an emphasis on animal efficiency. For example, enteric methane (CH_4_) represents an energy loss of 2–12 % of gross energy intake for individual large ruminants [1]. Measurements of enteric CH_4_ and carbon dioxide (CO_2_) emissions, and oxygen (O_2_) consumption can be used to estimate energy expenditures of cattle [2]. Additionally, enteric CH_4_ represents the largest source of greenhouse gas emissions from ruminant production systems [3] and mitigation of CH_4_ represents the most promising means to limit climate change in the short term [4,5]. Accurate gas flux estimates therefore provide important information when investigating means to improve the energetic efficiency and to reduce the greenhouse gas emissions of livestock production. Furthermore, accurate emission factors that are regionally and system specific are needed. This information is paramount for climate related goal setting by company’s within the livestock supply chain [6], national inventories required by the United Nations Framework Convention on Climate Change (UNFCCC) [7], and policy makers aiming to make climate related decisions. Ultimately, research to improve ruminant animal production efficiency and to quantify and reduce enteric CH_4_ are contingent on more accurately measuring gas fluxes from individual animals in actual grazing and confined feeding environments.

Prior efforts to measure gas fluxes in cattle include use of whole-body respiratory chambers for conducting indirect calorimetry estimates [8]. These whole-body systems place an animal inside an enclosed chamber that allows frequent measurement of individual animal gas fluxes. Stationary head box head-box systems [9] function similarly to the whole-body respiration chambers with only the animal's head enclosed. Although these two systems are considered the ‘gold standard’ of measuring gas fluxes [10], the problem is these systems are costly to build and are labor intensive, resulting in small sample sizes, and they remove cattle from their routine herd production environment.

Current techniques used to measure gas fluxes of ruminants in their production environment have been recently reviewed [11]. Briefly, the sulfur hexafluoride (SF_6_) tracer release technique was developed to quantify enteric methane (CH_4_) from unrestrained cattle in their production environment [12]. This technique inserts a SF_6_-containing bolus into the rumen with a known permeation (release) rate. The mixture of released gases (including the SF_6_ tracer) is captured inside a pre-evacuated canister that is secured to a harness on the animal's head and/or back. Concentrations of SF_6_ and CH_4_ within the canister are analyzed and daily CH_4_ emissions are calculated relative to the known quantity of SF_6_ released by the ruminal bolus. The SF_6_ technology is also labor intensive, making it difficult to measure many animals at a time. Additionally, increased required frequency of handling animals for this technique may impact the results. The SF_6_ method is also integrative, thus diurnal variations of CH_4_ emissions cannot be calculated, nor can CO_2_ and O_2_ fluxes be estimated.

The large ruminant GreenFeed system (**GFS**; C-Lock Inc., Rapid City, SD) is the next generation technology for measuring individual animal gas fluxes of methane (CH_4_), carbon dioxide (CO_2_), oxygen (O_2_), and hydrogen (H_2_) in production environments of both grazing and confined-fed cattle [13,14]. Small ruminant GFS are available that have detection capabilities below 10 g per day (CH_4_) for these animals’ smaller breath ’clouds'. The recommendations listed are specific for the large and small ruminant GFS. Small ruminant GFS are recommended for beef and dairy animals under six months of age, or for research involving small ruminants like sheep and goats. The GFS uses a real-time gas analyzer with a hood-like enclosure (see Fig. 2, Panel A, Item 3) where animal visits are incentivized (or baited) through a feeding setting. The GFS allows numerous unrestrained animals to be sampled by one system. Animal numbers measured for each GFS range from 20 to 60, thus requiring less labor and animal handling than the other techniques previously mentioned. In tie-stall or individual pen settings, the spot sampling offered by GFS does require manual labor to move systems among animals. The GFS can also be used in more extensive management systems such as grazing lands (pastures and rangelands) where animals are not frequently handled [15,16].

**Fig. 1:**
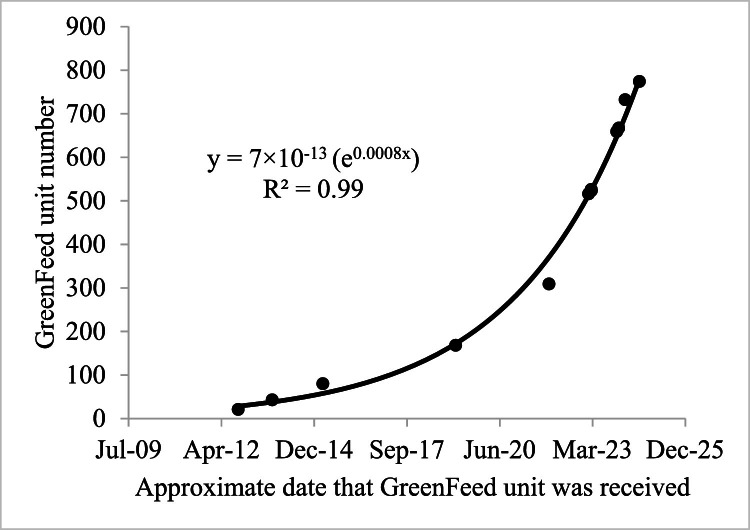
GreenFeed unit number (13 units ranging from #21 through 732) against date received, demonstrating that use of the GFS has exponentially increased (From C-Lock product log tracking process). This figure represents a subsample of GreenFeed units known to the authors.

The GFS has experienced rapid increases in deployments since their introduction in the mid 2010s. Currently, over 800 GFSs have been or currently in use (Fig. 1; C-Lock product log tracking process). This exponential increase in adoption (Fig. 1), necessitates a standardized procedure for experiments across different research groups. A prior review with recommendations for GFS was conducted eight years ago [14], with substantial methodological developments and experiential learning occurring recently that merits a more current set of recommendations. Therefore, our objective is to provide updated recommendations for using the GFS in large and small ruminant research and applied settings with contrasting animal types (beef and dairy) and production environments (grazing and confined fed systems). By standardizing GFS procedures, data generated from the GFS will be more comparable across experiments and laboratories and will provide increased accuracy in gas flux estimates. These are important goals as they will directly impact our capacity to conduct meta-analyses and for generating regionally and production system specific emission factors in support of national inventories.

## Method details

### The greenfeed system

The GFS is a modified open-circuit respiration chamber that functions similar to the head-box chamber [9]. Rather than restraining the animal in a metabolism stall and continuously measuring gas fluxes, estimates of daily gas fluxes are calculated from multiple, short-term visits (i.e., sample points) by individual animals to the GFS. Several studies have compared GFS for measuring respiration gas flux by cattle to other systems [10,14,[16], [17], [18], [19], [20]].

**Fig. 2:**
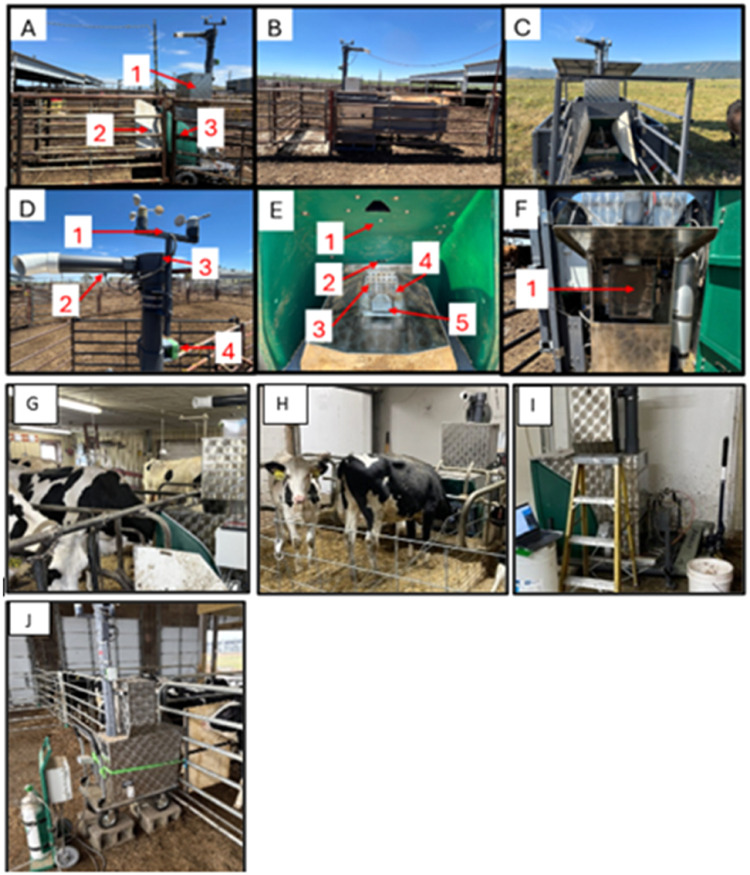
Pictures of GreenFeed unit types and components of the system. **Panel A:** a free stall unit, item 1 is the feed hopper, 2 is the wooden wind block, 3 is the GreenFeed hood; **Panel B:** a skid unit; **Panel C:** a pasture unit; **Panel D:** item 1 is the weather station measuring wind speed and direction, 2 is the air subsample line, 3 is where the fan is housed, 4 is the airflow rate sensor; **Panel E:** inside the GreenFeed hood, item 1 shows where the EID reader is located, 2 is the head proximity sensor, 3 is the feed pan with air intake manifold, 4 shows where the CO_2_ release system is inserted for CO_2_ recoveries, 5 shows where feed is dispensed; **Panel F:** the back of the GreenFeed showing where the primary air filter is located.; **Panel G:** the GreenFeed in a tie-stall setting with short airstack adaptation and tie-stall cart; **Panel H:** the GreenFeed in a tie-stall setting with short airstack adaptation and tie-stall cart with 6-month old dairy animals; **Panel I:** Tie-stall cart adaptation on large ruminant GreenFeed; **Panel J:** Small ruminant GreenFeed unit with 6-month old heifers.

Fig. 2 presents an overview of the GFS and its primary components. The GFS is designed and manufactured in variable configurations to meet requirements within the animal’s production environment (e.g., inside, outside, open pen, pasture, rangeland, freestall, tie-stall, etc.) and size and type of animal measured (e.g., small ruminant, large ruminant, young, mature, dairy, beef).

**Fig. 3:**
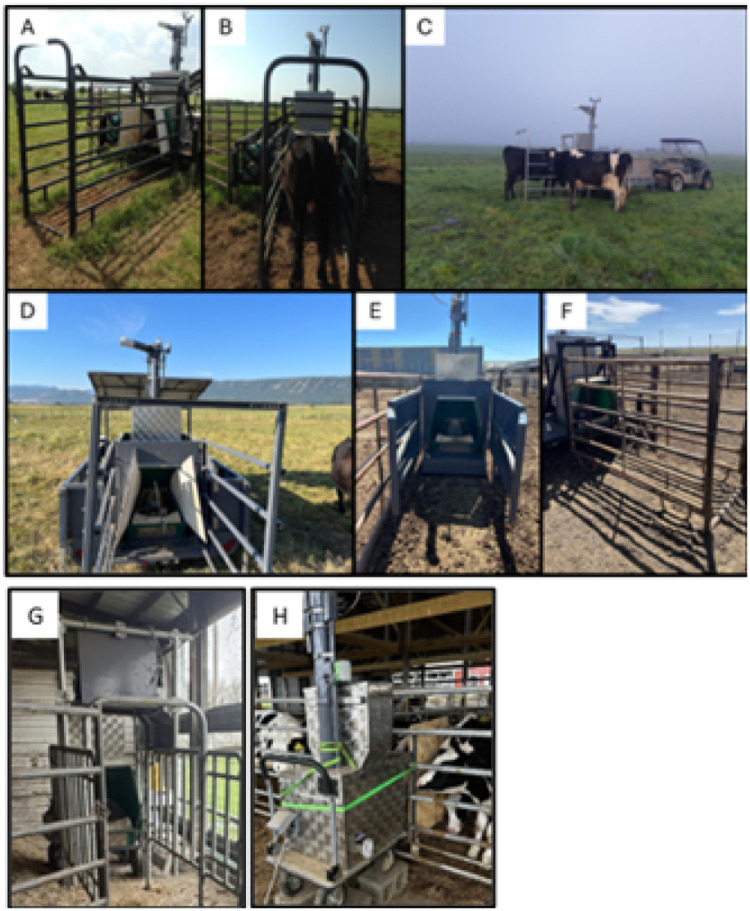
Different GreenFeed units and alley way set-ups that have been used. **Panels A, B**, and **C** show different alley set ups for two different pasture units. **Panel D** shows the alley set up, which is integrated into a newer pasture unit. **Panel E** shows the integrated alley for a skid unit. **Panel F** shows an example alley set up for a free stall unit. **Panel G** shows an example alley set-up for a free stall unit in a dairy freestall pen. **Panel H** shows an example of a small GreenFeed unit in a freestall dairy heifer barn.

In Fig. 3, Panel A represents a freestall unit (#309), Panel B a pasture unit (#659), Panel G a tie-stall unit (#527) with lactating cows, and panel H a tie-stall unit (#323) with prepubertal heifers. The GFS are sequentially numbered from the manufacture to identify the production order. Smartphone and tablet applications are used to connect with GFS real-time in the field to control unit operations and interfacing with the central data center information through this production numbering approach.

The pasture GFS are powered through solar panels with battery storage or 110v electric power. Power supply selection is dependent on the season of use, power requirements of the GFS (e.g., optional heating systems for cold environments), and availability of electricity (e.g., limited in remote locations). Access to the GFS by livestock should be limited to only the location for entry to the hood-like enclosure where data measurements are collected on individual animals. Experience indicates that free stall units, either large- or small-ruminant GFS, require additional protection in the form of gates or fencing around the GFS to prevent animal access to the GFS that may result in damage as well as facilitating single animal access to the hood-like enclosure to prevent multiple animals being sampled at the same time. Tie stall units do require a cart adaptation to house the GFS (Fig. 2, Panel I) with this cart and lifting mechanism providing for increased ease of maneuvering the GFS around corners in tightly spaced areas and raising it to allow easier animal access (Fig. 2, Panel G).

The GFS common components (see Fig. 2, Panel A) include item 1, a feed storage hopper, item 2, a wind block (free stall unit, wooden or plastic attachment) or integrated solid alley sides (skid or wheeled systems), item 3, a the hood-like enclosure where an individual animal places their head for emission measurement. Fig. 2, Panel D identifies the ‘stack’ of instrumentation used to collect environmental and emissions data. If using the unit in a tie-stall setting, an adapted, shorter stack may be required and installed to accommodate the ceiling clearance (Fig.1, Panel H). Panel D, Item 1 – a weather station, accounting for wind speed and direction when GFS are used outdoors; Item 2 – air sample collection tube for subsequent analysis of gas concentrations; Item 3 – housing for the fan that draws air around the animal’s head and through the system for gas collection; Item 4 - mass airflow sensor.

Fig. 2, Panel E describes the inside of the GFS hood. Panel E, Item 1 –radio- frequency identification (RFID) recorder; Item 2 – Animal head proximity sensor; Item 3 - feed pan with air intake manifold; Item 4 – location for the CO_2_ release system is inserted for CO_2_ recovery; Item 5 – location where feed is dispensed. Animals are trained to place their heads into the GFS hood by using a feed attractant. Access to the unit is established based on the experimental protocol and user-defined settings. Every animal has an electronic identification (EID) tag placed in either ear that is linked to a RFID, recording entry and exit times at each visit. The proximity sensor measures the animal’s head location when visiting the GFS. Air is drawn over the animal’s shoulders, past the muzzle, and through the feed pan to capture the gaseous emissions as bait is consumed and air samples are analyzed for specific gas concentrations.

**Fig. 4:**
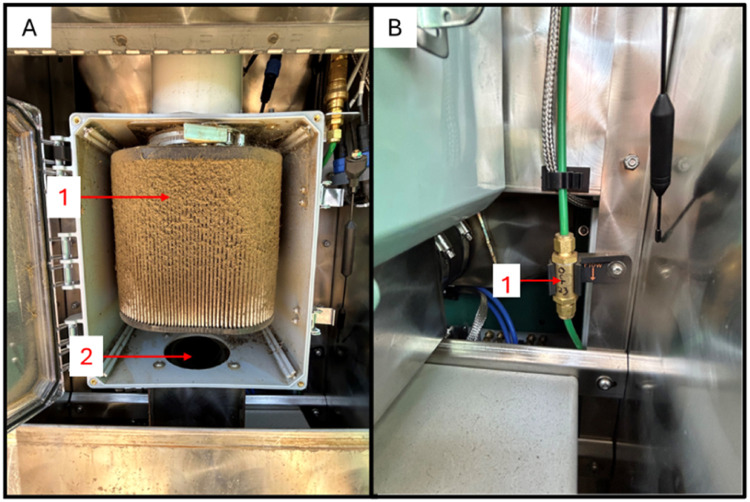
The primary air filter (A) and in-line sample filter (B).

Fig. 2, Panel F shows the back of the GFS, where the primary air filter is accessible. Fig. 4, Panel A shows a dirty primary air filter (Item 1) housed inside the plastic box located in the back of the GFS. Fig. 4, Panel A, Item 2 displays the direction of air flow from the GFS hood as it moves through the primary air filter, initially removing dust and large particles. In Fig. 4, Panel B is the in-line sample filter that provides another level of filtering following the subsampling of air occurring at the top of the PVC stack.

**Fig. 5:**
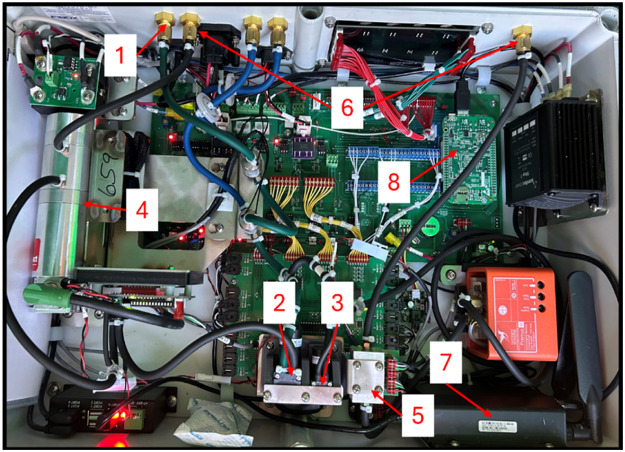
The electronics box which contains sample pumps, gas analyzers, and other electronic components. Item 1 shows where subsampled air enters the electronics box. Item 2 is the sample pump for the carbon dioxide (CO_2_) and methane (CH_4_) analyzer. Item 3 is the sample pump for the oxygen (O_2_) analyzer. Item 4 is the non-dispersive near-infrared CO_2_ and CH_4_ analyzer. Item 5 is the paramagnetic O_2_ analyzer. Item 6 shows where the gas flows from the sensor out of the electronics box. Item 7 is the cellular modem. Item 8 is the “beagle bone” providing Bluetooth capabilities.

Fig. 5 is a picture of the electronics box containing sample pumps, gas analyzers (Table 1), and other electrical components that operate the GFS. This box is located underneath the feed pan as shown in Fig. 2, Panel E, Item 3. Fig. 5, Item 1 displays where subsampled air enters the electronics box. Fig. 5, Item 2 is the sample pump that sends air sampled to the non-dispersive near-infrared CO_2_ and CH_4_ sensors (Item 4). Item 3 is the sample pump that sends air to the paramagnetic O_2_ sensor (Item 5). Depending on the gas analyzers installed, there may be more sample pumps and gas analyzers. Item 6 shows where air exits the electronics box after gas analysis by the sensors. Item 7 is the modem connecting the GFS to Wi-Fi. The SIM card is obtained by a local mobile phone provider and inserted into the modem. Item 8 is a BeagleBone (a type of a minicomputer) that provides Bluetooth capabilities for the GFS enabling operation of the system through the “Control Feed” app.

**Table 1:** Sensor specifications used in GreenFeed units.

Gas analyzed	Sensor Type	Measurement Range (ppm)	Detectable Limits	Error	Accuracy
CH_4_	Nondispersive infrared absorbance^1^	0 to 4000	Min. 30 g/d	3 %	3 %
CH_4_	Tunable Diode Laser^2^	0 to 40,000	1 g/d	0.3 %	0.3 %
CO_2_	Nondispersive infrared absorbance	0 to 20,000 ppm	0 to 10,000	< 1 %	0.5 %
O_2_	Paramagnetic	0 to 1000,000	0 to 100 %	< ± 0.1 %	NA
H_2_	Electrochemical	0 to 100	<1 ppm	NA	NA

1 Nondispersive infrared absorbance used on GreenFeed units <272.

2 Tunable Diode Laser used on GreenFeed units 272 and greater.

**Fig. 6:**
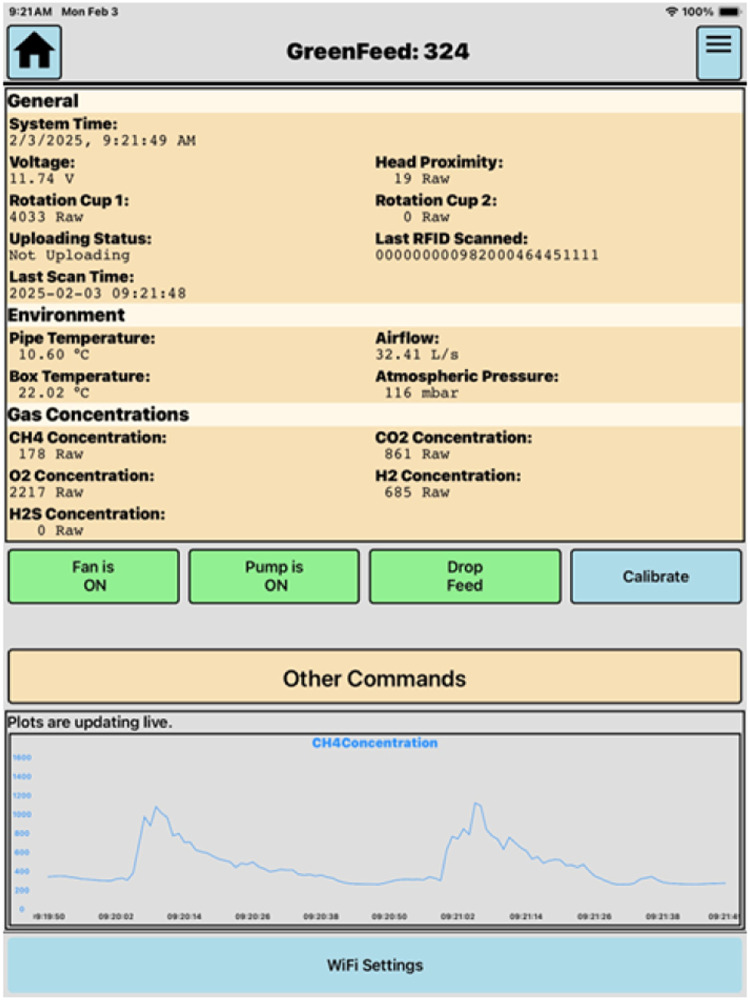
Screenshot of tablet during spot sampling process with live raw methane being display.

It is recommended having a designated tablet per unit when spot sampling in tie-stall to use the “Control Feed” app. In a tie-stall sampling, the app aids in monitoring each unit for proper head proximity and monitoring raw CH_4_ emissions to aim for a minimum of three eructation sessions per sample time point (Fig. 6). In our experience, lactating cattle require around between 1 and 2 min between eructation bouts. The head proximity detects the muzzle distance from the air intake manifold and used to evaluate proper proximity to the unit for accurate gas flux calculations. A value of 800 is default and required for the start of feeding to occur. The head proximity is also used to determine which time periods gas fluxes should be calculated.

### Recommended procedures

The sections below outline GFS use recommendations based on the manufacturer, experiential and experimental evidence by the author group and greater research community. The processes, practices, and procedures within provide a background and starting point for research conducted using GFS. Recommendations may not be applicable in every experimental setting, nor should they be considered the only approach to achieving your research goals. If your sampling situation is unique, the support staff at C-Lock, Inc. will provide operational and setting recommendations and consider consulting with other experienced researchers.

### General maintenance

Guidance for the general maintenance of the GFS can be found online (URL: https://docs.c-lockinc.com, accessed 07/10/2025).

### Air filters

Air flowrate is a critical component of effective gaseous emissions estimation. Airflow through the feed pan collects dander from the animal, dust from the environment, and feed offered. The primary air filter (Fig. 2, Panel F) is located at the back of the unit. Accumulation of dirt (Fig. 4, Panel A, Item 1) can significantly reduce airflow. Manufacturer recommendations indicate that the primary air filter be replaced when airflow drops below 26 L/s in large ruminant GFS and 10 L/s in small ruminant GFS. Experimental support for this threshold is discussed in greater detail below. When replacing the primary air filter, we recommend scooping out any dust buildup under the filter then vacuuming the box completely after each filter change. The in-line air sample filter (Fig. 4, Panel B) is recommended to be replaced at least annually. The in-line filter is directional, and installers should ensure that the arrow on the filter points in the direction of airflow (down and toward the electronic box).

### Calibration and CO_2_ recovery

When utilizing the GFS, calibration factors are required for two values being detected by GFS, the concentration (ppm) of each gas and airflow (L/s) through the system. We recommend contacting C-Lock Inc. Customer Support for specific instructions for calibration of gas concentration sensors for each GFS. Proper and timely calibration and re-calibration of sensors ensure accurate results and GFS under serial number 483 have auto-calibration with zero and span gas tanks, typically conducted automatically every 3 days.

GreenFeed units 483 and greater are equipped with auto recovery systems that only include a span gas tank, where C-Lock Customer Support establishes timelines for remote calibration, as frequently as daily based on research needs. Both systems serve the same purpose for targeted gas concentrations used to recalibrate the sensors. We recommend weekly calibrations while in operation and after the primary air filter is changed. Manual calibrations using standard-gas filled foil gas sample bags can also be conducted.

The standard calibration procedure is used to convert the raw voltage changes noted by the sensors to concentration (ppm) for each gas measured. A two-point concentration is used for calibrating the system. The standard calibration initially releases a zero gas containing 20 % O_2_ and 80 % N_2_. After the release of the zero gas, a span gas or calibration gas containing 50 – 1500 ppm CH_4_, 0.5 to 1 % CO_2_, 21 % O_2_, and 10 ppm H_2_ in pure nitrogen is released to the gas sensors. Each gas runs separately for 60 s, pausing between releases, in total running 5 min. To generate a correction factor, the difference between analyzed concentration of the span gas is compared to the concentration of these gases measured by the sensor.

When using the Auto-Recovery System, the gas calibration occurs via a mass flow controller with the air velocity transmitter, thereby allowing the process of manual CO_2_ recovery and auto-calibration to be combined. The span tank contained 3.5 % CH_4_, 11.5 % CO_2_, and 400 ppm H_2_ balanced by pure N_2_. The gases are released at 10L/min for 1 min to release 3246 g/d CH_4_, 361 g/d CO_2_, and 0.5 g/d H_2_. To generate a correction factor, the difference between analyzed concentration of the span gas is compared to the concentration of these gases measured by the sensor.

Standard calibrations are performed prior to beginning an experiment to establish correction factors. Calibrations should be compared against previous standard calibrations ensuring the change in correction factor does not exceed 5 %. If a greater than 5 % difference occurs, C-Lock should be contacted to run the standard calibration and evaluate the unit functionality. Troubleshooting is conducted remotely by C-Lock, Inc. unless the performance issue is not resolved.

**Fig. 7:**
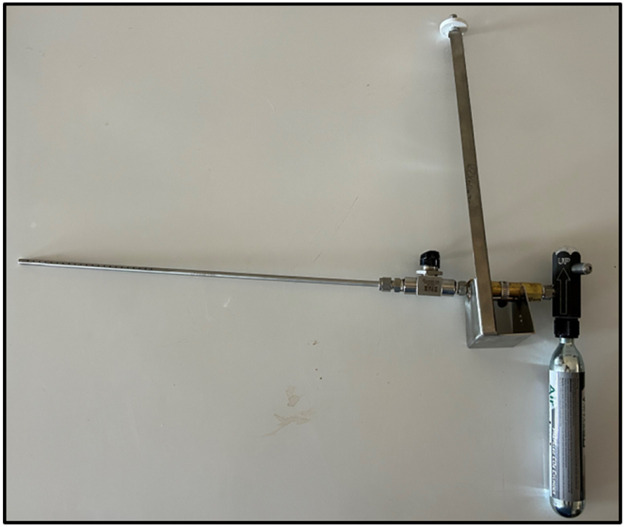
The CO_2_ release system for CO_2_ recoveries.

Note: The largest potential for error during this process is in weighing the CO_2_ release system at the start and end of each CO_2_ release event. This should be done with at least to the nearest 0.1 g, avoiding wind at this level of resolution; any amount of wind may result in scale fluctuations, making obtaining an accurate weight difficult. Recoveries should be within ± 5 % of each other and typically are 95 to 105 %. Options to avoid wind include weighing indoors or using an enclosed boxed that houses the scale and the CO_2_ recovery system can be weighed. Accumulation of ice on the canister is also a very common cause of error. Wiping CO_2_ canisters with ethanol aids in rapidly melting the ice and timely weighing of the canister.

The CO_2_ recovery method assesses gas capture by the GFS by comparing the total mass of CO_2_ released (based on before and after weights of the CO_2_ release system) to the amount measured in the GFS. It is best practice to report the average and standard deviation CO_2_ recovery rate. Calculating recovery rates are used to assess the gaseous capture rate of the machine and used as an indirect validation of sensor calibrations. Carbon dioxide recoveries can be calculated less frequently than standard calibrations. We recommend completing CO_2_ recovery rate calibrations at least monthly and should be done following replacement of the primary airfilter and following concentration calibrations of the gas analyzers. Carbon dioxide recoveries use a CO_2_ release system (Fig. 6), with a 90 g CO_2_ canister attached (e.g., JT 90 g prefilled paintball CO_2_ tank; Montreal, QC) Fig. 7.

The steps for CO_2_ recoveries are the following:

1. Weigh the CO_2_ release system with the CO_2_ cylinder attached for an initial weight and record the start time.
2. Insert the assembled CO_2_ release system into the air-intake manifold of the feed pan, where a washer has been welded (Fig. 2, panel E, Item 4).
3. Open the system valve to release CO_2_ for approximately 3 min and record the exact time.
4. Repeat the process 3 more times with 2 min between each release and record the exact time
  a. When temperatures decrease below 4 °C, increase the time per release by 3 to 4 min.
5. After each CO_2_ release, the system is weighed and the mass of CO_2_ release can be calculated by the difference between initial and final weight of the CO_2_ release system plus canister.
6. Log into your GFS account, select the data tab (Fig. 8, Item 1), then select the CO_2_ recovery test tab (Item 2).

**Fig. 8:**
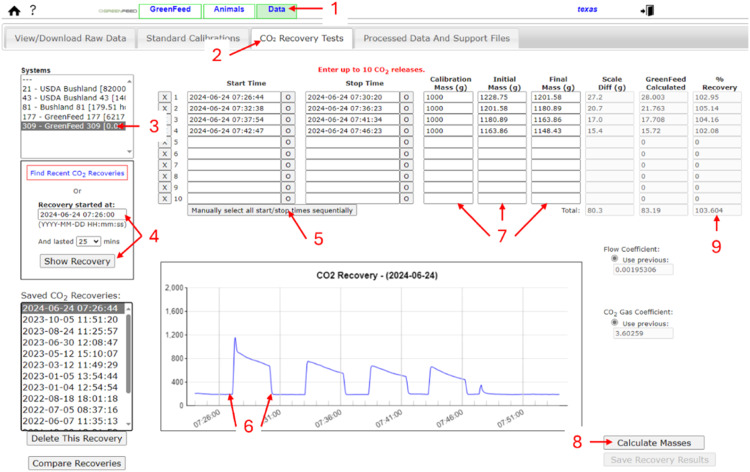
Web-interface for determining carbon dioxide recovery rates. Items 1 and 2 show the tabs to select to reach the carbon dioxide (CO_2_) recovery web interface. Item 3 shows where to select the appropriate GreenFeed unit. Item 4 shows where to initially identify the recovery by entering the start time and duration. Item 5 shows where to select so that start and end time of each CO_2_ release can be selected on the graph. Item 6 shows an example of where you would click on the graph to identify the start and end time of each CO_2_ release. Item 7 shows the columns to enter the weights measured in the field. Selecting the button identified by item 8 tells the unit to calculate GreenFeed estimated mass of CO_2_ released. Item 9 shows the calculated CO_2_ recovery, comparing the mass of CO_2_ release determined by manually weighing with the GreenFeed estimated CO_2_ release.
7. Select the appropriate GFS from the available list of systems (Fig. 8, Item 3).
8. Enter the correct start date and time including an appropriate duration on either side of the recovery and select “Show Recovery” (Fig. 8, Item 4). The graph should display the CO_2_ recovery event with clear peaks where the CO_2_ was released.
9. Select “Manually select all start/stop time sequentially” (Fig. 8, Item 5) and an ellipsis (i.e., …) will appear in the start time column for the first recovery event. Manually click on the Fig. and the beginning and the end of each CO_2_ release peak (Fig. 8, Item 6) and start and stop times will automatically appear in the cells.
10. Enter the initial and final weight of this CO_2_ release system plus CO_2_ cylinder (Fig. 8, Item 7) and select “Calculate masses” (Fig. 8, Item 8).

he “ % Recovery” column will automatically fill and you can check that the average is within ± 5 % + (Fig. 8, Item 9). Note: it is good practice to ask C-Lock, Inc. for support to check CO_2_ recoveries. Recoveries may decrease by 10 % when temperatures drop below 4 °C.

To facilitate automatic calibration and CO_2_ recovery processes, new GFS have a “gas mass flow controller” and auto-recovery system installed that can accurately release gases from cylinders with known gas concentrations. This new approach is undergoing validation to replace manual CO_2_ recovery. The current recommendation is to maintain manual processes until validation is complete. Table 2 has descriptions of the calibration techniques, the coefficient being calculated and method of calculations.

**Table 2:** Calibration methods summary**.**

Method of Calibration	What is Being Calculated	Method of Calculation
Standard Calibration	Gas Coefficients	Concentration sensor responses to gases
CO_2_ Recovery	Gas capture rate	Comparing total mass injected vs. mass captured by system
Auto-Recovery	Gas Coefficients and capture rate	Comparing mass injection rate vs. Mass capture rate

## Training, number of animals, greenfeed set-up, and bait feeds

### Training to the greenfeed

Introducing novel items to cattle, such as a GFS, may alter animal behavior, questioning measurement reliability [21]. Therefore, positive training is critical to establishing the routine of regular visits to the hood of the GFS. Visits are essential to gather enough observations per animal within treatment in each experimental setting. During early training, we recommend adding the pelleted supplement or some other attractant into the feed pan a few times a day to stimulate interest. Other feeds, including distillers dried grains with solubles, soybean meal, or ground corn in minimal (<100 g) amounts can be used as attractants during training and placed by hand into the feed pan. However, they are not recommended for use in the feed bin during sample collection because of the high concentration of fines, which would increase the frequency that primary air filters would need to be swapped. Further discussion regarding bait selection is addressed below.

Our experiences indicate that training animals in drylot pens, where animal proximity to the unit are controlled, aid in the training process and routine visits, even if the units will eventually be used in a pasture or rangeland setting. In an initial research study conducted in a 40-ha tall-grass prairie research pasture, researchers reported of the 25 candidates started with the GFS, only 14 cattle had adequate GFS visitation frequency of at to be included in CH_4_ analysis or approximately 56 % [22]. In subsequent experiments, cattle were trained to a GFS in a dry-lot pen, a high proportion (∼80 %) of available cattle visited with adequate frequency [[23], [24], [25], [26], [27], [28]].

Cattle experience initial hesitancy visiting the GFS if entering via an alley and/or if wing blocks near the hood or not present. Where feasible, we recommend removing the blocks early in the training process and then re-installing. In newer GFS, the alley may be integrated into the frame, making wind blocks or alley removal more challenging (Fig. 3, Panel D – pasture unit; Panel E – skid unit). In these situations, we recommend initially opening the alley to the widest setting and narrowing the alley overtime to reduce the risk of multiple animals attempting to enter and interfere with access during the experiment. Additionally, in certain grazing situations using the pasture trailer, it may be recommended to leave the alley in the raised position or to unbolt from the trailer, leaving the GFS entirely open while animals become accustomed to using the units. The visit frequency can be monitored and once animals have learned to use the equipment, the alley can be lowered and narrowed.

Previous studies report that 20 to 30 % of beef cattle may not adequately adapt to the GFS [14,22]. If possible, researchers should train more animals than required for a given experiment. Identification and selection of individual animals for experimental use can be determined by data generated from visit frequency during training. Based on similarity in visit criteria to the GFS, individual visit data may increase uniformity of assigning treatments to animals. However, authors caution the potential to create treatment bias. For example, when using random allocation from a trained population, it was reported only one animal visited the GFS in the control treatment group [22]. Accordingly, investigators may consider stratifying animals based on pre-trial GFS visitation and then randomly assigning animals to treatment within each stratification.

The following exploration leads us to recommend initially programming the GFS to dispense feed every 24 s while the animal is visiting the GFS, with a minimum time between visits where feed is delivered every 4 h in a feedlot or pasture setting. After approximately one week, panels used to form the alley can be added, the width of the stationary alley adjusted, and wind blocks reattached to meet experimental settings. Flexibility in decisions regarding training timelines is essential, as each group of cattle is different in their rearing background, stage of life, temperament, and environmental conditions including the season and weather events that can require training adjustments. Experience indicates that 2–8 weeks of training may be necessary, and we recommend planning for a 4-week training period in most scenarios. Eight weeks of training may be required in pasture situations on expansive landscapes.

In a tie-stall barn situation, a setting where spot sampling is used, Hristov et al. [13] detailed adaptation to the GFS and training dairy cows to the machine. Briefly, the machine is introduced visually, with eventual placement of the unit in front of the cow. Recently, in studies with 75 or more mid-lactation Holstein cows, after first topdressing the bait feed on the animals’ ration in the feed bunk, three separate daily sessions of 2 to 5 min each trained over 97 % of animals [29]. Occasionally, feed drops are set at 30 s during initial training and increased to 45 s during collection to minimize dry matter intake (DMI) at the GFS. Similar to previous reports [13], when dairy heifers 6 months of age were introduced to the GFS in a tie-stall system or individual pen set-up, the unit was placed within view of the animals for at least 2 days before allowing animals to enter the measurement chamber. The feeder chime and feed drop are activated at least twice daily during that time where the animal can hear the feed drops. Consider providing the feed typically fed to the animals as a bait feed through the GFS to encourage approaches due to familiarity [30]. In a freestall barn, emissions of late lactation dairy cows previously trained to the GFS in a tie-stall barn were compared to those that had not been trained. After both groups received similar training over a 2-d period by being brought to the machine every 24 h, no difference occurred in visits to the GFS or average daily emissions [26]. Results indicate blocking for previous training is not required in a freestall barn situation.

### Number of animals per GFS

The number of animals per GFS depends on their proximity to the system and animal stocking density. In pasture experiments, 20 to 25 animals per GFS is normally recommended. However, in extensive pastures with low stocking density, 20 animals might be optimal versus up to 25 in small pastures with greater stocking density. In confinement settings, reports of success with 27 animals per GFS are demonstrated, with a note that traditional feed bunk space limited their opportunity to evaluate greater animal numbers [26,28]. In pasture situations, research has reported placing the GFS unit near the water tank. Given the gregarious nature of cattle, the number of animals per unit also depends on the balance between watering and time in the GFS, with the herd’s willingness to remain while the last individuals receive their bait.

Other researchers have successfully evaluated 50 animals per GFS in a confined beef setting (Sara Place, Colorado State University, personal communication). Published and anecdotal information supports allocation of 40 to 50 animals per GFS in confined experimental settings, with numbers adjusted based on training success and temperament of the cattle within the context of experimental conditions. Recent studies with lactating dairy cows in a freestall setting demonstrated one unit supporting data collection on 60 cows over a 7 to 8-week timeframe.

In tie-stall barns, animal numbers are dependent on management routines (e.g., milking and feeding times). Therefore, 20 to 25 animals allow for up to 3.5 h of spot sampling by a single person, assuming 5 min GFS sampling per cow and 2 min rest period between each animal. Feed in feedbunks should be removed before placing the unit in front of the animal. Depending on proximity, one person may run 2 units simultaneously, allowing for staggering of animals and increasing numbers of possible animals to collect from 40 to 50.

### User defined settings

The goal is to have cattle remain with their head in the hood of the GFS or ≥3 min per visit and to have visits spread as evenly as possible throughout the day to help account for diurnal variation in instantaneous gas flux. To achieve these goals, the researcher can program the number of drops per visit, the number of seconds between drops, a minimum time among visits, and the maximum number of visits per day.

We typically utilize 24 s between each drop with 8 drops per visit [[25], [26], [27], [28]] 30 s between each drop with 6 drops per visit [[22], [23], [24]]. For research studies more concerned with the proportion of DMI and associated nutrients from consumption of bait offered in the GFS, establishing a 30 s interval and 6 drops per visit will, in theory, reduce bait DMI by 25 % relative to a 24 s interval with 8 drops per visit. Importantly, Guter et al. [31] determined an effect of bait dispense interval on estimates of CH_4_ or CO_2_ emissions and concluded that if the cattle remained for >3-min per visit, the number of drops and the interval between drops did not affect respiration gas flux estimates.

In tie-stall barns, or when spot sampling is used, 5 to 6 drops with 45 s between each drop can be used [29,32]. If using the large ruminant GFS in a tie-stall studies with dairy heifers 6 months of age to 18 months of age, settings should be adjusted to 8 drops with 45 s between drops to target 5 to 10 min of sampling data or consider shortening the interval to 30 s as described above [30]. Due to the head proximity of younger animals, more time in the GFS may be required to meet the 3-min minimum gas collection threshold per visit for quality of data during processing. The small and large ruminant GFS used in a freestall barn uses 800 (no units) head proximity as default, with 30 s feed drop intervals, and 8 drops per setting up to 6 times per day. The head proximity indicates the presence of an animal at the unit. With younger animals, head proximity settings may need to be decreased to 600 from the default of 800 in the C-Lock interface to allow for proper dispensing of feed given their smaller muzzle. However, C-Lock will need be notified to adjust for calculations based on final results where 600 is considered good data versus 800.

As the number of allowable visits per day is increased, DMI from bait feeds will likely increase. In high DMI animal experiments (e.g., lactating dairy cows, mid- to late-finisher beef cattle) we suggest increasing the number of allowable visits and decreasing the minimum time between visits. This strategy would allow the opportunity for more visits per day in some experimental settings, without greatly increasing the proportion of pellet intake to total DMI. For example, a dairy cow with an expected DMI of the basal TMR of 25 kg per day, could receive 0.5-kg of GFS concentrate per day and this would only represent 2 % of their total DMI, whereas a grazing beef steer with a daily DMI of 6 kg per d of the basal forage consuming the same amount of GFS concentrate per day would have 7.7 % of their total daily DMI from the pellets. In a freestall experiment, we allowed 8 visits per day with a minimum of 3 h between visits, where DMI was expected to be 14 kg of alfalfa (*Medicago sativa*) baleage per day during the dry period and then 18 kg per day of a perennial ryegrass (*Lolium perenne*) pasture during the lactation phase [27]. However, in this experiment there were still only 2 visits per cow per day in the prepartum period and 0.9 visits per head per day in the postpartum period, indicating allowing 8 visits per day did not translate into greater GFS use [27]. Other freestyle research on late lactation dairy cows demonstrated allowing 6 visits per day or minimal 4 h between GFS visits also resulted in an average of two visits per day [32].

In other experiments with growing stocker beef cattle grazing warm-season perennial grass pastures, cool-season annual forage pastures, or fed finishing diets in confinement, we allowed four visits per day with 4 h of minimum duration between visits [[23], [24], [25], [26],^28^]. In one study, [22] we programmed the GFS to allow for 3 visits per day with 6 h of minimum time between visits. This experiment had the worst number of usable visits per animal (and in total) with several animals not meeting the assigned threshold number of “good” total visits per animal (i.e., 30 visits; [33,34]). It is unknown if these programmed settings caused reduced GFS visits or if it was attributed to poor training and less palatable bait feed. Based on their findings, we recommend programming the GFS to allow for 4 visits per day with 4 h of minimum duration between visits as we had success with this program previously.

### Bait feeds and nutritional considerations

**Fig. 9:**
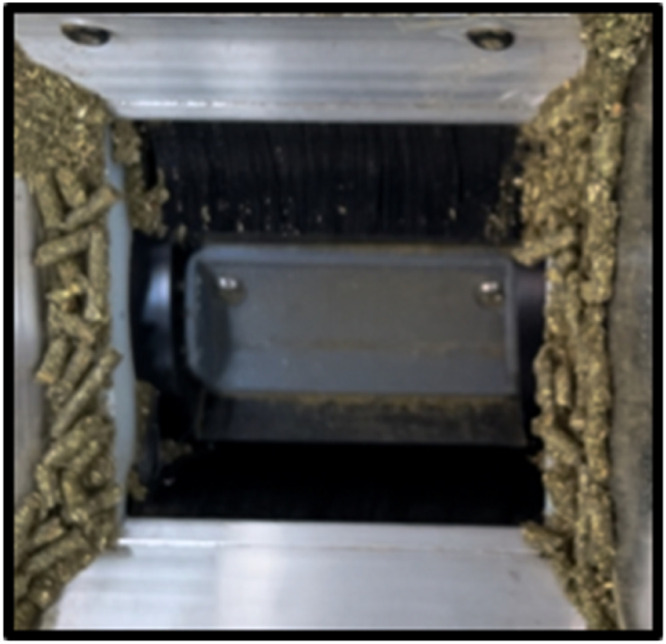
The cylinder and cup are responsible for dispensing the pelleted bait supplement.

Overall, the feed provided in the GFS is used to attract cattle to the unit and keep the animal’s head within the measurement hood for a sufficient amount of time. However, bait feed can also offer composition and palatability attributes, or supplement nutrients that differ from the basal diet, which could confound the results of nutritional research. The type of bait feed is based on the experimental objective of the research and any nutrient constraints. Typically, a pelleted or texturized feed is optimal for consistency of the physical feed and mass delivered at each cup rotation. The mass of feed delivered depends on the volume that can fit into a cup embedded in a rotating cylinder (Fig. 9).

Examples of successful pelleted supplements and their use scenarios from GFS experiments include: a) 97 % soybean meal and 3 % molasses pellet used in tall grass prairie pastures throughout the summer; b) alfalfa pellets offered to cattle grazing in mixed grass prairie pastures; c) wheat middlings offered to cattle grazing winter wheat; d) a manufactured, multi-feedstuff commercial alpaca and llama pellet offered to dairy cows fed with access to alfalfa baleage and grazing perennial ryegrass pastures; or e) a custom formulated low protein (12 % or less) commercial pellet. The feed should be 7 mm in diameter or less and 21 mm or less in length (C-Lock, Inc.).

Pelleted and texturized feeds are used to reduce dust that will clog the air filter, allowing the unit to maintain airflow above 26 L/s in the large-ruminant GFS or 10 L/s in a small-ruminant GFS. Providing bait feeds with greater levels of fines or dust particles will require more frequent air filter cleaning and replacement and decrease consistency in the mass delivered at each rotation that will impact expected DMI. Also, increases in fines may reduce the nutrient availability by increasing variability of nutrient intakes. A pellet durability index over 75 had <2 % fines, although multiple factors such as conditioning temperature, starch, protein, and oil quantity and form can influence the durability and nutritive properties [35,36]. Experience indicates that feed quality and proportion of fines is influenced by formulation and product handling. For pelleted supplements with excess starch, fat, and too little heat during the pelleting process, fines will increase. If persistent dust occurs, pellets may require reformulation or additional liquid added in the case of texturized bait feeds. After feed delivery, storing in a moisture-free space is essential to avoiding clumping of feed, which can jam the equipment, and slow visits to the unit.

Understanding the impact of pellet diameter, and texturized feed particle size on average weight and its variation is important for two reasons. First, researchers will have increased confidence in offered pelleted DMI from the GFS unit when a consistency dispensed feed is used. Second, if the research objective aims to use the feed to dose an animal with an external or internal marker to measure fecal output, estimate DMI, or test a supplement, then a smaller variation between drops will reduce variability in the average dose rate [25].

The mass of feed offered at each visit is required to estimate daily DMI for each animal over a defined period. The user controls the maximum number of feed drops within a visit, the maximum number of visits per day, and the time interval between visits. The number of dispenses per day is downloaded to estimate the daily DMI contribution from the GFS. The cup that dispenses the feed attractant is a fixed dimension, where the diameter of the feed influences the mass of each drop from the cup.

Calculating the average and standard deviation of individual cup weights is recommended based on averaging 20 individual drops. Previously when this technique was used, smaller diameter pellets occupied greater volume and consistency between drops. For example, the average drop weight of a 4.8-mm diameter pellet (avg. 32.5 g, std. dev. 1.6 g, CV 4.9 %) was heavier and less variable when compared with a 6.4-mm diameter pellet (avg. 30.1 g, std dev. 3.0 g, CV 10.0 %). The feed cup in the small ruminant GFS is smaller and has greater variability when dispensing a texturized dairy heifer grower (avg. 6.5 g, std. dev. 1.0 g, CV 15.4 %). The same texturized dairy heifer grower dispensed in a large ruminant GFS had lower variability (avg. 35.5 g, std. dev. 1.5 g, CV 4.2 %).

The amount of bait feed offered at the GFS is based on user settings to ensure a 3-min collection time for data analysis and may represent a large proportion of total DMI. The contribution of bait feed to the total diet depends on the basal DMI. Beef cattle grazing tall grass prairie pastures in late summer with an initial body weight of 279 ± 8 kg consumed 2.1 to 4.5 % of their total DMI as GFS pellets. In finishing beef cattle, pellet intake from the GFS represented 4.0 % to 6.4 % of total DMI [26,28]. Prepartum dairy cows limit-fed alfalfa silage had 2.6 % to 4.7 % of total DMI composed of GFS pellets, whereas the same set of cows grazing pastures dominated by perennial ryegrass and white clover (*Trifolium repens*) had 1.7 % to 2.7 % of their DMI from the pellets [27]. A critical assumption made when calculating the proportion of feed intake from the GFS is that all the feed dispensed to an animal is consumed. While <1 % refusals are typically noted in tie-stall studies with lactating dairy cows, refusals recorded at the end of spot sampling prepubertal dairy heifers reported a range from 2 to 30 % refusals of texturized feed based on an average of 7 drops, averaging 35.5 g per drop, 45 s apart [30].

Researchers must consider the nutritive characteristics of the expected DMI at the GFS and impact on experimental outcomes based on the animal’s nutrient requirements. Data on total drops per animal can be downloaded from the ‘Animals’ tab by selecting the ‘Animal Statistics’, selecting the GFS of choice, date range of interest, and selecting the ‘Download’ function. The composition of the bait feed directly impacts the nutrient intake of the animal. For example, Proctor et al. [28] conducted an experiment to determine how the feeding level of ruminal degradable protein (RDP) influenced nitrogen excretion and enteric CH_4_ emissions. The treatments were formulated to provide 87 %, 100 %, and 113 %, of total digestible nutrient (TDN) allowable microbial crude protein (MCP) synthesis (i.e., RDP required) resulting in a low RDP, neutral RDP, and high RDP treatment, respectively [37]. Based on the total DMI (base diet + pellet supplement), the in situ analyzed supply of RDP resulted in an RDP balance of 104.3 %, 111.2 %, and 122.2 % of TDN allowable MCP synthesis. In contrast, without accounting for the GFS pellet intake, RDP balances were calculated as 98.3 %, 111.8 % and 119.8 % for the low, neutral, and high RDP treatments and closer to target levels [37]. While intakes represented 6.4 %, 5.4 %, and 6.0 % of DMI for the low, neutral and high RDP treatments, the consumed alfalfa pellets at the GFS unit provided 12.0 %, 8.8 %, and 9.1 % of RDP intake. In retrospect, Proctor [37] acknowledged the need to have identified a bait feed with lower RDP content.

In grazing scenarios, the choice of pellet attractant can induce unwanted supplementation effects, introducing confounding errors into a given experiment [38]. Issues arise when the GFS attractant contrasts with pasture nutritive value and alters the consumption of pasture DMI by additive, substitutive, or combined effects [38]. Pellet attractants should be similar in nutrient composition to the pasture as reasonably possible. For instance, when Tifton 85 Bermudagrass (*Cynodon dactylon*) hay was pelletized, which was similar in nutrient composition to the *Brachiaria brizantha* pastures where beef steers were grazing [39]. By adding vanilla extract to the pelleted hay, the steers were successfully trained to the GFS equipment, eliminating the need to use other supplements. In this example, a protein, starch, or high fat pellet or texturized bait feeds would change total nutrient intake of the animals and, as a result, alter nutrient digestibility or animal production.

In tie-stall barn experiments where all cows are sampled at once including randomized complete block, Latin-Square or other designs, bait feed can be included in the common total mixed ration (**TMR**) during times where GFS samples are not being collected. During sampling periods, the bait feed that would normally be provided through the TMR was removed and provided through the GFS. Assuming cows consume all offered feed by the GFS, TMR DMI should not be impacted. Similarly, when conducting experiments using the GFS and automatic milking systems (**AMS**), using the same pellet fed through the AMS and the GFS allows adjustments to the total pellet offered through the AMS to be adjusted based on the feed offered at the GFS. Making these adjustments will not alter the contribution of concentrate to forage ratio in the overall formulated diet. For example, if targeting 1 kg intake through the GFS, remove the same amount at each milk the volume level in the AMS feed allotment.

## Data handling

### Preprocessing

When a user downloads the processed data from the C-Lock inc. website, one row of data is obtained for each individual visit to the GFS (further described in the subsequent section). From this, researchers must preprocess the data to arrive at an estimate of gas fluxes for each animal at the researchers’ preplanned temporal scale. A researcher could use the dataset with each individual visit directly to the GFS to explore treatment effects using a mixed model, and some laboratory groups do use this approach [40,41]. However, a single estimate, averaged over a preplanned period, for each animal is necessary for subsequent calculations, such as CH_4_ yield (g CH_4_ per unit of DMI) and intensity (g CH_4_ per unit of production). These data are then used as independent variables for equations, such as metabolic heat production [2,42] to determine statistical associations, such as correlations between CH_4_ and DMI or to identify low and/or high CH_4_ emitting animals [43]. The following sections will describe common methods for the use and conversion of raw data collected as individual rows for individual animal GFS visits to an estimate of gaseous emissions for an individual animal and recommendations for data preprocessing.

### Accessing data and important columns

Data from each GFS visit is available to download from the C-Lock, Inc. website (URL: https://ext.c-lockinc.com/home) once logged into a user account. After logging in, select the “Data” tab and then the “Processed Data and Support Files” tab. There will be a list of Excel files that can be downloaded. This list will also show the file size and the last time that the file was modified. User can use the time that the file was last modified as an indication of which file contains the most up-to-date data. After downloading and opening the appropriate file, there will be a series of Excel sheets. The sheet of primary interest is the “Events”, which will have 25 columns. The column names may be different, depending on the GFS. The most important columns include: the animal EID, the user defined animal visual ID, the GFS number, the start and end times of the visit, the visit duration, the visit hour of the day (ranging from 0 to 23.99), and the average airflow (L/s).

### Initial data cleaning

Initial data cleaning is based on a determination of adequate airflow for each visit logged with the removal of visits with inadequate airflow. Adequate airflow is necessary to ensure complete capture of the breath cloud while the animal’s head remains in the GFS hood. The experimentally determined threshold for adequate airflow is a minimum of 26 L/s. In analysis of suboptimal airflows, Gunter et al. [31] reported that from 10 to 26 L/s there is a linear increase in estimated CO_2_ and CH_4_ emissions as airflow increased and airflows above 26 L/s there is a plateau, indicating complete capture of the cattle’s breath cloud. These results indicate an underestimation of gas emissions below 26 L/s with large-ruminant GFS and hypothetically 10 L/s with the small-ruminant GFS although it has not been measured. The research findings coincide with the recommendations of C-Lock, Inc., and the recommendation is to remove any visits with average airflow rates below 26 L/s from subsequent analysis. The primary reason for suboptimal airflows is a clogged air filter, emphasizing the need for regular cleaning of the air filter at an interval determined by the experimental conditions.

The initial data cleaning protocol establishes a time threshold for the minimum duration of a visit to the GFS hood. C-Lock, Inc. GFS software algorithms automatically remove any visit <2 min in duration from the reported data. To investigate the impact of a visit duration <2 min on CO_2_ and CH_4_ emissions, 24,195 visits recorded from four experiments [[22], [23], [24],^28^] were obtained from C-Lock, Inc. Across the four experiments, 47.1 % of observations were < 2 min in duration [34]. On average across the four experiments, a visit duration of <1 min had 24.2 % lower CO_2_ emissions, 44.6 % lower O_2_ consumption, and 69.8 % lower CH_4_ emission estimates when compared with emissions estimates from visits ≥ 3 min in duration. When visits were ≥ 1 min and < 2 min in duration, results indicated an 11.5 % lower CO_2_ emission, 12.5 % lower O_2_ consumption, and 22.9 % lower CH_4_ emission than those ≥3 min. Additionally, visits ≥ 2 min and < 3 min in duration had 7.3 % lower CO_2_ emissions, 5.4 % lower O_2_ consumption, and 4.9 % lower CH_4_ emission estimates when compared with estimates derived from visits ≥3 min in duration.

Analyses of data from published reports indicate that in pen-based and pasture-based studies 40 visits of ≥ 2 min in duration per animal, or 30 visits of ≥ 3 min in duration per animal are required to provide adequate emission estimates [34]. This analysis demonstrates a 25 % reduction in required visits per animal per day if visit duration is lengthened to ≥ 3 min [40]. The aforementioned findings agree closely with [33] who determined that for 2-min visit duration threshold 45 GFS unit visits are required and for a 3-min visit duration threshold 30 GFS visits per animal would be required. Other research reported that for a 2-min visit duration threshold a minimum of 40 visits per animal were required albeit visit requirements to meet a 3-min visit duration threshold were not accessed [44]. In an experiment, it was determined that for a 3-min visit duration threshold achieving target visit number would require approximately 20 d in a grazing experiment and 13 d in a confined feeding experiment [34]. Whereas, if a 2-min visit duration threshold was used, approximately 25 d would be needed in a grazing study and 15 d in a confined experiment. Knowing that emission estimates are lower when visit duration is between 2 and 3 min, and the number of visits required per animal is greater for a minimum 2-min duration, we recommend excluding all data for visits with <3 min duration and including only data from animals with 30 or more visit records for further analysis. In tie-stall settings where dairy cattle are monitored throughout the data collection, 8 to 10 spot samples throughout are adequate for analysis. These sampling periods are spaced 5 – 7 h apart based on milking times and management routines over a 3 to 4 d period. In a freestall setting, dairy cattle visited an average of 20 times over a 12 day period [32].

### Outliers

Outliers and extreme values can occur with all data, including gas flux measured by the GFS. The research community suggests removal of data points that exceed the variable’s third quartile plus three times the interquartile range [45]. Previous research [46] summarized the number and (percentage) of outlier data points in five GFS data sets. Using this threshold, there were 3 (0.2 %) [22], 5 (0.4 %) [23], 1 (0.1 %) [24], 0 (0.0 %) [26] and 34 (0.4 %) [28] observations removed. Removal of the observations reduced estimated CH_4_ emissions by 0.2, 0.4, 0.3, 0.0, and 3.7 g/d [[17], [18], [19],^22^,^23^]. Based on these five experiments where the number and percentage (≤ 0.4 %) of total data points within an experiment were removed, removal of extreme values did not change the CH_4_ estimates to a large degree. The frequency (number and percentage) of data points considered outliers should be investigated and consistently reported in findings. If a large proportion of measurements are determined to be outlier data points, other sources of error should be identified by additional review of the recording and reporting process.

### Methods for arriving at a single animal estimate

This section will describe arriving at a gas flux estimate for each individual animal. These next approaches occur after you have removed visits with <26 L/s airflow rate in large ruminant GFS and 7/s in small ruminant GFS, and those that were <3 min in duration. Perhaps the most used method is simple arithmetic averaging. The arithmetic averaging approach simply averages across all GFS visits within animal. The primary equation used to calculate gas mass flux based on the recoveries for determining coefficients and correction factors. In general, the mass flux is the product of the gas concentration with the airflow measurement and the dimensional coefficient determined from measurements conditions and second order effects (Table 2). There is some concern that this approach does not adequately account for diurnal variation, especially in instances where cattle preferentially visit the GFS at a particular time of the day. To better account for diurnal variation, time bin averaging was proposed [47]. This method first averages visits into 6 (4-h interval) to 8 (3-h interval) time bins for each animal. Averages are calculated across the time bins within animal. This method weights each time bin equally, accounting for variation in the number of visits within a time bin. A potential issue with time bin averaging is that time bins with low visit numbers may be over-inflated [34].

There is a known diurnal variation in instantaneous CH_4_ emissions from ruminants across various production systems [15,46,48]. The degree to which emissions vary throughout the day appear to be diet and production system related. It was determined that beef cattle fed a finishing diet had a much larger diurnal variation in CH_4_ emissions than grazing beef cattle [46]. In pastoral systems, maximum CH_4_ emissions appear to typically occur during the night (1800–0600 h) and minimum CH_4_ estimates occur sometime during the day [15,46]. However, this diurnal pattern is practically the opposite for beef cattle fed finishing diet, where maximum CH_4_ emissions typically occur 2 to 3 h after feeding and minimum CH_4_ emissions typically occurred immediately prior to feeding [46]. In pastured cattle, the maximum CH_4_ estimates were 21 % to 45 % greater than the minimum estimates, whereas the maximum estimates of CH_4_ emissions were 77 % to 109 % greater than minimum CH_4_ estimates in beef cattle fed a finishing diet. In both tie-stall and freestall barns for lactating dairy cattle, the diurnal patterns reflect the initial feed delivery and the number of times the feed is pushed up to the feedbunk throughout the day. The greatest increase is 1 – 4 h following fresh feed delivery, with the lowest levels being within 3 h before fresh feed delivery [29,49].

Recently, an alternative technique to account for diurnal variation when preprocessing GFS data was proposed [46]. This approach utilizes mixed model analyses to generate a least-squares means (LSMEANS) for each individual animal. The mixed model we used included each gas as the dependent variable, animal ID as the fixed effect, airflow, and visit duration as covariates (removed if P–value > 0.10), and included date and hour of day nested within animal ID as random effects. The LSMEANS approach also provides a standard error of the mean for each animal’s estimate. Researchers can weigh each animal’s set of observations within the statistical model, based on the standard error of the mean for that estimate as described previously [50]. The researcher can determine if including this weighting procedure improves the model by reducing residual standard deviation [46]. Using the standard error of the mean to weigh each animal’s estimates resulted in lower residual standard deviation in 2 of the 5 experiments used for the investigation [46]. Time-bin averaging of CH_4_ estimates were 3.9 % greater than the arithmetic average in pastoral based experiments [46]. The LSMEANS approach provided estimates of CH_4_ emissions that averaged 0.1 % greater than the arithmetic average estimates for those same pastoral based studies [46]. In finishing studies, time-bin and LSMEANS estimates averaged 8.7 % and 7.2 % lower, respectively [46]. These findings suggest that accounting for diurnal variation is more important in finishing trials than in grazing studies. This is likely due to finishing trials having greater diurnal variation in feed intake and hence emissions than grazing studies [15,48]. Despite it being less important to employ techniques to account for diurnal variation in pasture studies, we still recommend using them to maintain consistency in data processing methods across experiments.

Based on our findings, time bin averaging increased the coefficient of variation by 9.1 % relative to arithmetic averaging and 9.9 % relative to the LSMEANS approach. This increase in unexplained error would require an additional 4-animal observations per treatment (22 % increase) to detect a 10 % treatment difference (assuming a beta of 0.20 and alpha of 0.05) compared with the LSMEANS approach [46]. Our experience indicates the LSMEANS approach accounts for diurnal variation with similar and lower residual standard deviation relative to arithmetic and time bin averaging, respectively. We recommend researchers generate individual animal estimates using the described LSMEANS approach [46].

### Final data considerations

Application performance interfaces (**API**) are also being created to aid in processing in real time of GFS data utilizing R markdown in combination with C-Lock platform [51,52]. Further statistical packages in R have been created to aid in daily and final processing of GFS data, visits to the GFS, and feed offered while allowing for parameters to be user-defined to accommodate their systems [51]. Using APIs during on-going research may be useful when monitoring GFS performance and data quality, although similar functions can be achieved directly through the C-Lock interface and is necessary to download GFS feed drop data.

## Conclusions

The objective of this methods article was to provide a recommended protocol for utilizing the GFS to measure enteric gas flux of cattle in confinement and pasture-based systems. Also, considerations were suggested for bait feed and data processing. Throughout the manuscript, we have provided recommendations based on experiential and experimental evidence. A summary of these recommendations can be seen in Box 1. For those approaches based on experiential evidence, we realize that others may utilize different approaches that are equally or more effective or improve data quality compared to those we recommend. In that case, we do not suggest users alter their existing protocols. However, based on experimental evidence, where applicable, our suggestions may benefit all users. These include the importance of feed composition, both physical and nutritive to ensure consistency and feed delivery, experimental settings at the GFS, impact on nutrient intake, data preprocessing methods, outlier detection, and data cleaning. As the GFS becomes more widely used there becomes a greater need for standardized protocol so experiments from various lab groups are repeatable and comparable to each other the protocols listed herein represent our suggestion for such a standardized protocol based on cattle type and management. However, reviewers and editors need to ensure that researchers adequately describe the protocols utilized for their experiments, especially for data preprocessing techniques.

**Box 1.** Summary of the experiential and experimentally determined recommendations when using the GreenFeed.

**Table:** 

Situation	Recommendation
Experiential Recommendations:	
Training in Dry-lot or freestall	Start training with 20 % more cattle than the study requires Train Cattle in a dry-lot pen Allocate 4-weeks for training Begin with panels and wooden wind-blocks remove (if possible) As cattle progress slowly add these back
Training for Spot-sampling or tie-stall	Place unit in animal housing area prior to training Acclimate animals to the GreenFeed bait feed Place on the manger or top-dress animal’s feed Have 3, 5 min training sessions for each cow prior to starting the experiment
Animal Allocations per GreenFeed:	20–25 cattle per GreenFeed in pasture Likely that more can be used at higher stocking densities and less at lower stocking densities 40–50 cattle per GreenFeed in confinement studies (e.g., feedlot) For tie-stall, number of cows per GreenFeed is time limited Spot samples may only be collected over 3-hour period 20–25 cows may be measured assuming 5 min of sample collection and 2 min of rest per cow
Bait Feed:	Use as small diameter of pellet as possible Will have more uniform drop weights Need to collect and weigh individual drops so that a mean ± standard deviation can be determined We recommend weighing 20 drops Consider how GreenFeed pellets will impact total intake and influence objectives of the experiment GreenFeed pellets can represent 1.5 % to 6.5 % of total dry matter intake (depending on basal diet intake) If spot sampling in a tie-stall, include 1.5 % of ration dry matter as GreenFeed pellets Remove during sampling time
User Defined Visit Settings:	Set the GreenFeed to dispense 5–8 drops per visit Set drops 24–45 s apart Set 4–6 minimum time between allowable visits Set 4–6 total allowable visits per day If concerned with GreenFeed pellet intake then set system to allow 6 drops per visit, with 30 s between drops, 4 allowable visits and 6 h between visits If less concerned with GreenFeed pellet intake then you can change all settings to more frequent times In tie-stall, allow 5–6 drops per visit, with 45 s between drops (allows for 3–5 min of good data) Younger dairy animals (6–12 mo) may require more and more frequent drops
Experimental Recommendations:	
Outliers:	Extreme data points do not significantly influence the overall estimate of gas fluxes Based on our previously conducted experiments If extreme data are significantly influencing estimates, then likely a larger issue is occurring
Airflow:	For large animal units, remove visits with <26 L/s average airflow rate For small ruminant units, remove visits with <10 L/s average airflow rate
Minimum visit duration thresholds:	Remove visits <3 min in duration Visits < 3 min have considerably lower extimates than those ≥ 3 min When ≥ 3 min are used as the threshold, will require 30 visits per head If ≥2 min is used as the threshold, will require 40 visits per head The added visits from the lower threshold do not appear to account for the increased observations needed Requires more experimental days
Data preprocessing:	Fit all visit data to a mixed effects model, where gas flux estimate is the dependent variable and animal ID is the fixed effects Include data and hour of the day by animal ID as random effect Generate least-squares means (LSMEANS) for each animal ID to provide that animal’s estimated gas emission or consumption Explore the appropriateness of weighting the observations in subsequent statistical analyses using the standard error of the mean generated by the LSMEANS procedure

## Method validation

The methods outlined here are based on the authors’ experiences obtained over the last decade and also experimentally supported. The experimentally supported recommendations have been published in previous peer-reviewed manuscripts and the appropriate references are provided throughout the text.

## Limitations

### Operational considerations

Securing the GFS from damage from animals is vital to the structural integrity of the equipment and to the quality of the data collected. Depending on the type of unit, special considerations must be made for protecting the equipment. Newer model trailers for pasture use have improved where most unit sections are blocked off by steel panels and parts are out of reach from most grazing animals. Similarly in free-stall barns, the GFS area is blocked with gates, fencing, or side panels. In improved pastures, temporary electric fencing can be used to prevent animals from damaging the GFS trailer. This would allow for easy movement in rotational stocking systems, where animals are frequently allocated to new paddocks. If electric mobile fencing is unavailable, corral fence panels may be set up to create a perimeter around the GFS.

Additional opportunities exist to identify improved methodology for utilization of GFS for research purposes. In pasture and rangeland settings, attractants co-located with GFS to help encourage animal visitation including water source, salt/mineral supplemental tubs need to be evaluated. The influence of these co-located items on visitation has only been anecdotally evaluated and more powerful evaluations are needed. The influence of multiple water sources, shade and lighting throughout the day, forage quality, and impacts of larger extensive pastures on GFS visits also needs to be elucidated [53]. Additionally, the impact of moving grazing dairy cattle to and from the pasture twice a day for milking, especially if cattle spend time in the barnyard consuming concentrates or stored forages (and therefore would spend time away from a pasture-based GFS), must be considered. Continued research is needed on how emission intensity and gas fluxes change throughout the life in multiple ruminant species. Also, the variation in gas flux and animal efficiency associated with being moved between management systems such as from pasture to feedlot in beef or from pasture to confinement with dairy heifers need to be elucidated.

## Ethics statements

**Animal Care and Use Committee from each respective institution and research facilities approved all animal procedures from research reported in this methods manuscript.**

## Acknowledgments

All contributors who do not meet the criteria for authorship should be listed in an acknowledgments section.

Research was funded by the USDA-Agricultural Research Service. USDA is an equal opportunity provider, employer, and lender. Any use of trade, firm, or product names is for descriptive purposes only and does not imply endorsement by the U.S. Government.

## Declaration of competing interest

The authors declare that they have no known competing financial interests or personal relationships that could have appeared to influence the work reported in this paper.

## Supplementary material and/or additional information [OPTIONAL]

Data will be made available upon request.

## Data Availability

Data will be made available on request.

## References

[1] Johnson K.A., Johnson D.E. (1995). Methane emissions from cattle. J. Anim. Sci..

[2] Brouwer E., Blaxter K.L. (1965). Energy Metabolism, Proceeding of the 3rd Symposium, European Association for Animal Production.

[3] EPA, Inventory of U.S. Greenhouse gas emissions and sinks: 1990-2022, U.S. Environmental Protection Agency, Washington D. C., 2024. https://www.epa.gov/system/files/documents/2024-04/us-ghg-inventory-2024-main-text_04-18-2024.pdf.

[4] Thompson L.R., Beck M.R., Larson H., Rowntree J.E., Place S.E., Stackhouse-Lawson K.R. (2025). Is climate neutral possible for the U.S. beef and dairy sectors?. Front. Sustain. Food Syst..

[5] UNFCCC, Kyoto protocol to the United Nations Framework Convention on Climate Change, Kyoto, 1997. https://unfccc.int/cop4/resource/docs/cop3/l07a01.pdf.

[6] Kleiber M. (1935). The California apparatus for respiration trials with large animals. Hilgardia.

[7] Place S.E., Pan Y., Zhao Y., Mitloehner F.M. (2011). Construction and operation of a ventilated hood system for measuring greenhouse gas and volatile organic compound emissions from cattle. Animals.

[8] Hill J., McSweeney C., Wright A.D.G., Bishop-Hurley G., Kalantar-zadeh K. (2016). Measuring methane production from ruminants. Trends Biotechnol..

[9] Hristov A.N., Bannink A., Battelli M., Belanche A., Cajarville Sanz M.C., Fernandez-Turren G., Garcia F., Jonker A., Kenny D.A., Lind V., Meale S.J., Meo Zilio D., Muñoz C., Pacheco D., Peiren N., Ramin M., Rapetti L., Schwarm A., Stergiadis S., Theodoridou K., Ungerfeld E.M., van Gastelen S., Yáñez-Ruiz D.R., Waters S.M., Lund P. (2025). Feed additives for methane mitigation: recommendations for testing enteric methane-mitigating feed additives in ruminant studies. J. Dairy. Sci..

[10] Johnson K., Huyler M., Westberg H., Lamb B., Zimmerman P. (1994). Measurement of methane emissions from ruminant livestock using a SF6 tracer technique. Environ. Sci. Technol..

[11] Hristov A.N., Oh J., Giallongo F., Frederick T., Weeks H., Zimmerman P.R., Harper M.T., Hristova R.A., Zimmerman R.S., Branco A.F. (2015). The use of an automated system (GreenFeed) to monitor enteric methane and carbon dioxide emissions from ruminant animals. J. Visualiz. Experiments 2015.

[12] Gunter S.A., Beck M.R. (2018). Measuring the respiratory gas exchange by grazing cattle using an automated, open-circuit gas quantification system. Transl. Anim. Sci..

[13] Gunter S.A., Bradford J.A. (2015).

[14] Dorich C.D., Varner R.K., Pereira A.B.D., Martineau R., Soder K.J., Brito A.F. (2015). Short communication: use of a portable, automated, open-circuit gas quantification system and the sulfur hexafluoride tracer technique for measuring enteric methane emissions in Holstein cows fed ad libitum or restricted. J. Dairy. Sci..

[15] Hammond K.J., Humphries D.J., Crompton L.A., Green C., Reynolds C.K. (2015). Methane emissions from cattle: estimates from short-term measurements using a GreenFeed system compared with measurements obtained using respiration chambers or sulphur hexafluoride tracer. Anim. Feed. Sci. Technol..

[16] Huhtanen P., Cabezas-Garcia E.H., Utsumi S., Zimmerman S. (2015). Comparison of methods to determine methane emissions from dairy cows in farm conditions. J. Dairy. Sci..

[17] McGinn S.M., Coulombe J.F., Beauchemin K.A. (2021). Technical note: validation of the GreenFeed system for measuring enteric gas emissions from cattle. J. Anim. Sci..

[18] Ma X., Räisänen S.E., Wang K., Amelchanka S., Giller K., Islam M.Z., Li Y., Peng R., Reichenbach M., Serviento A.M., Sun X., Niu M. (2024). Evaluating GreenFeed and respiration chambers for daily and intraday measurements of enteric gaseous exchange in dairy cows housed in tiestalls. J. Dairy. Sci..

[19] Hemsworth P.H., Price E.O., Borgwardt R. (1996). Behavioural responses of domestic pigs and cattle to humans and novel stimuli. P.H. Hemsworth et Ul./Appl. Animal Behav. Sci..

[20] Beck M.R., Thompson L.R., White J.E., Williams G.D., Place S.E., Moffet C.A., Gunter S.A., Reuter R.R. (2018). Whole cottonseed supplementation improves performance and reduces methane emission intensity of grazing beef steers. Prof. Anim. Sci..

[21] Beck M.R., Thompson L.R., Williams G.D., Place S.E., Gunter S.A., Reuter R.R. (2019). Fat supplements differing in physical form improve performance but divergently influence methane emissions of grazing beef cattle. Anim. Feed. Sci. Technol..

[22] Thompson L.R., Beck M.R., Gunter S.A., Williams G.D., Place S.E., Reuter R.R. (2019). An energy and monensin supplement reduces methane emission intensity of stocker cattle grazing winter wheat. Appl. Animal Sci..

[23] Beck M.R., Gunter S.A., Moffet C.A., Reuter R.R. (2021). Technical note: using an automated head chamber system to administer an external marker to estimate fecal output by grazing beef cattle. J. Anim. Sci..

[24] Beck M.R., Proctor J.A., Kasuske Z., Smith J.K., Gouvêa V.N., Lockard C., Min B., Brauer D. (2023). Effects of replacing steam-flaked corn with increasing levels of malted barley in a finishing ration on feed intake, growth performance, and enteric methane emissions of beef steers. Appl. Animal Sci..

[25] Beck M.R., Garrett K., Fleming A.E., Maxwell T.M.R., Greer A.W., Bunt C.R., Olejar K.J., Jonker A., Dynes R., Gregorini P. (2022). Effects of Lactobacillus fermented plant products on dairy cow health, production, and environmental impact. Anim. Feed. Sci. Technol..

[26] Proctor J.A., Smith J.K., Long N.S., Gunter S.A., Gouvêa V.N., Beck M.R. (2024). Utilizing gas flux from automated head chamber systems to estimate dietary energy values for beef cattle fed a finishing diet. J. Anim. Sci..

[27] Dittrich B.I., French E.A., Kammann E.M., Breunig M.K., Kalscheur K.F., Franco J.G., White H.M., Kononoff P. (2025). 2025 ADSA - Annual Meeting.

[28] Wu Z., Cunha T.O., Hernandez L.L., French E.A., Kononof P. (2024). 2024 ADSA - Annual Meeting.

[29] Gunter S.A., Bradford J.A., Moffet C.A. (2017). Effects of mass airflow rate through an open-circuit gas quantification system when measuring carbon emissions. J. Anim. Sci..

[30] FitzGerald T.J., French E.A., Kononof P. (2024). 2024 - ADSA Annual Meeting, American Dairy Science Association, West Palm Beach.

[31] Arthur P.F., Barchia I.M., Weber C., Bird-Gardiner T., Donoghue K.A., Herd R.M., Hegarty R.S. (2017). Optimizing test procedures for estimating daily methane and carbon dioxide emissions in cattle using short-term breath measures. J. Anim. Sci..

[32] Beck M.R., Thompson L.R., Proctor J.A., Reuter R.R., Gunter S.A. (2024). Recommendations on visit duration and sample number requirements for an automated head chamber system. J. Anim. Sci..

[33] T. Winowiski, Feed Pelleting Reference Guide Measuring the physical quality of pellets, 2005. https://img.feedstrategy.com/files/base/wattglobalmedia/all/document/2019/09/fs.5-20_Measuring_the_physical_quality_of_pellets.pdf (accessed June 26, 2025).

[34] Kertz A.F., Darcy B.K., Prewitt L.R. (1981). Eating rate of lactating cows fed four physical forms of the same grain ration. J. Dairy. Sci..

[35] Proctor J.A. (2023).

[36] J.E. Huston, F. Rouquette, W.C. Ellis, H. Lippke, T.D.A. Forbes, Supplementation of Grazing Beef Cattle, College Station, TX, n.d.

[37] Mombach M.A., de Carvalho P., da S. Cabral L., de A.R. Rodrigues R., Torres R.C., Pereira D.H., e Pedreira B.C. (2018). Attractants for automated emission measurement (Greenfeed®) in pasture-based systems. Revista Brasileira de Zootecnia.

[38] Waghorn G.C., Jonker A., MacDonald K.A. (2016). Measuring methane from grazing dairy cows using GreenFeed. Anim. Prod. Sci..

[39] Jonker A., Scobie D., Dynes R., Edwards G., De Klein C., Hague H., McAuliffe R., Taylor A., Knight T., Waghorn G. (2017). Feeding diets with fodder beet decreased methane emissions from dry and lactating dairy cows in grazing systems. Anim. Prod. Sci..

[40] Kaufmann L.D., Münger A., Rérat M., Junghans P., Görs S., Metges C.C., Dohme-Meier F. (2011). Energy expenditure of grazing cows and cows fed grass indoors as determined by the 13C bicarbonate dilution technique using an automatic blood sampling system. J. Dairy. Sci..

[41] Smith P.E., Waters S.M., Kenny D.A., Kirwan S.F., Conroy S., Kelly A.K. (2021). Effect of divergence in residual methane emissions on feed intake and efficiency, growth and carcass performance, and indices of rumen fermentation and methane emissions in finishing beef cattle. J. Anim. Sci..

[42] Dressler E.A., Bormann J.M., Weaber R.L., Rolf M.M. (2023). Characterization of the number of spot samples required for quantification of gas fluxes and metabolic heat production from grazing beef cows using a GreenFeed. J. Anim. Sci..

[43] Tedeschi L.O. (2022). ASAS-NANP symposium: mathematical modeling in animal nutrition: the progression of data analytics and artificial intelligence in support of sustainable development in animal science. J. Anim. Sci..

[44] Beck M.R., Thompson L.R., Moffet C.A., Reuter R.R., Gunter S.A. (2025). Method: comparing averaging methods for gas flux data generated by automated head chamber systems. Animal - Open Space.

[45] Manafiazar G., Zimmerman S., Basarab J.A. (2017). Repeatability and variability of short-term spot measurement of methane and carbon dioxide emissions from beef cattle using GreenFeed emissions monitoring system. Can. J. Anim. Sci..

[46] Hales K.E., Cole N.A. (2017). Hourly methane production in finishing steers fed at different levels of dry matter intake. J. Anim. Sci..

[47] Nelson D.J., Kalscheur K.F., French E.A., Gunter S.A., Salmi A., Cunningham S., Simpson E., Graham M., Hamilton S., Gardner L., Kononof P. (2024). 2024 ADSA - Annual Meeting, American Dairy Science Association, West Palm Beach, Florida.

[48] St-Pierre N.R. (2001). Integrating quantitative findings from multiple studies using mixed model methodology. J. Dairy. Sci..

[49] Martinez-Boggio G., Lutz P., Harrison M., Weigel K.A., Peñagaricano F. (2025). greenfeedr: an R package for processing and reporting GreenFeed data. JDS. Commun..

[50] Brennan J.R., Parsons I.L., Harrison M., Menendez H.M. (2024). Development of an application programming interface to automate downloading and processing of precision livestock data. Transl. Anim. Sci..

[51] Raynor E.J., Schilling-hazlett A., Place S.E., Martinez J.V., Thompson L.R., Johnston M.K., Jorns T.R., Beck M.R., Kuehn L.A., Derner J.D., Stackhouse-lawson K.R. (2024). Snapshot of enteric methane emissions from stocker cattle grazing extensive semiarid rangelands. Rangel. Ecol. Manage.

